# An Integrative Approach to the Identification of Arabidopsis and Rice Genes Involved in Xylan and Secondary Wall Development

**DOI:** 10.1371/journal.pone.0015481

**Published:** 2010-11-23

**Authors:** Ai Oikawa, Hiren J. Joshi, Emilie A. Rennie, Berit Ebert, Chithra Manisseri, Joshua L. Heazlewood, Henrik Vibe Scheller

**Affiliations:** 1 Feedstocks Division, Joint BioEnergy Institute, Emeryville, California, United States of America; 2 Physical Biosciences Division, Lawrence Berkeley National Laboratory, Berkeley, California, United States of America; 3 Department of Plant & Microbial Biology, University of California, Berkeley, California, United States of America; University of Massachusetts Amherst, United States of America

## Abstract

Xylans constitute the major non-cellulosic component of plant biomass. Xylan biosynthesis is particularly pronounced in cells with secondary walls, implying that the synthesis network consists of a set of highly expressed genes in such cells. To improve the understanding of xylan biosynthesis, we performed a comparative analysis of co-expression networks between Arabidopsis and rice as reference species with different wall types. Many co-expressed genes were represented by orthologs in both species, which implies common biological features, while some gene families were only found in one of the species, and therefore likely to be related to differences in their cell walls. To predict the subcellular location of the identified proteins, we developed a new method, PFANTOM (plant protein family information-based predictor for endomembrane), which was shown to perform better for proteins in the endomembrane system than other available prediction methods. Based on the combined approach of co-expression and predicted cellular localization, we propose a model for Arabidopsis and rice xylan synthesis in the Golgi apparatus and signaling from plasma membrane to nucleus for secondary cell wall differentiation. As an experimental validation of the model, we show that an Arabidopsis mutant in the *PGSIP1* gene encoding one of the Golgi localized candidate proteins has a highly decreased content of glucuronic acid in secondary cell walls and substantially reduced xylan glucuronosyltransferase activity.

## Introduction

Plant cell walls are complex structures, predominantly composed of polysaccharides. Secondary walls develop in some cell types after the termination of cell expansion, and these walls usually contain lignin in addition to polysaccharides. The polysaccharides in secondary walls are largely represented by cellulose and hemicelluloses, particularly xylans. Pectin and other hemicelluloses, e.g. mannans and xyloglucans are much less abundant in secondary walls. For a recent review of hemicellulose structure and function, see Scheller and Ulvskov [1]. Xylans have a backbone of 1,4-linked β-xylosyl residues, some of which are substituted with single glucuronosyl (GlcA), 4-*O*-methyl-GlcA, and arabinofuranosyl residues. Furthermore, the xylose residues can be acetylated at O-2 and/or O-3, and in Poales the arabinofuranosyl residues can be feruloylated at O-5. More complex side chains can also be present, and the structural patterns vary both between species and tissues. Secondary walls in angiosperms contain xylan as the major hemicellulose, and this xylan generally has little or no arabinose and a high acetate content. Grass xylans tend to have more arabinose, and no arabinose has been detected in xylan from Arabidopsis. Xylans from different dicot and gymnosperm species, including Arabidopsis, have been shown to contain the complex structure β-d-Xyl-(1→4)-β-d-Xyl-(1→3)-α-l-Rha-(1→2)-α-d-GalA-(1→4)-d-Xyl at the reducing end [2], [3], [4]. Such structures are yet to be reported in grasses.

Pectin and hemicelluloses are synthesized in Golgi vesicles by glycosyltransferases (GTs) which use nucleotide sugars as donor substrates. The understanding of this biosynthesis is still rather limited, but multi-membrane-spanning enzymes belonging the Cellulose Synthase Like (CSL) family of proteins have been shown to synthesize β-1,4-linked backbones of mannans and glucomannans and be involved in biosynthesis of mixed linkage glucans and xyloglucan backbones. In contrast, the backbone of pectic homogalacturonan and sidechains of hemicelluloses and pectins seem to be synthesized by other families of GTs that are Type II membrane proteins. Recent reviews describe biosynthesis of hemicelluloses and pectin [1], [5]–[10]. Despite the abundance of xylans and their importance in wood, animal feed and food, little was known until recently about the genes required for xylan biosynthesis. In Arabidopsis, several genes involved in the formation of the secondary wall have been identified by screening for irregular xylem (*irx*) mutants and analysis of genes co-expressed with genes already shown to be involved in secondary wall formation [11], [12]. Several of the *irx* mutants are affected in genes encoding Type II membrane GTs that appear to be involved in xylan biosynthesis. These genes include members of families GT8 (*PARVUS*, *IRX8*), GT43 (*IRX9* and *IRX14*), and GT47 (*FRA8* also known as *IRX7*, and *IRX10*) [4], [13], [14]. The corresponding mutants have decreased xylan in stems, and an increase in the proportion of 4-*O*-Me-GlcA side branches relative to the non-methylated GlcA [13], [15]. Further biochemical analysis of the xylan reducing end structure and xylan chain length suggested that FRA8, IRX8 and PARVUS are involved in the synthesis of the reducing end structure, whereas IRX9, IRX10 and IRX14 may function in xylan backbone chain elongation [4], [13]–[15]. None of these proteins have had their biochemical activity demonstrated, but nevertheless the evidence that they are somehow involved in xylan biosynthesis is strong. The β-1,4-linked backbone of xylan led many to expect that CSL proteins would be responsible for synthesis of the backbone, but this seems highly unlikely as there is no candidate CSL family available for such an activity.

Co-expression analysis of genes is a method to identify candidate proteins involved in the same biological process, including proteins that function together in a complex. Along with the accumulation of microarray datasets, transcriptome co-expression analysis has proven to be a powerful tool for identifying regulatory relationships in the transcriptional networks of model organisms, including *Escherichia coli* [16], yeast [17] and Arabidopsis [18]. While Arabidopsis is well established as the primary model species in plant biology, rice is quickly gaining popularity as a model organism. In addition to the availability of substantial genetic, molecular, and genomic resources, two features make rice attractive as a reference species: it represents distinct monocots and is a crop species. In an important transcriptional study, Mitchell et al. [19] compared EST data available for members of the Poaceae with transcriptional data for dicots. Based on this data they proposed candidates of GT families involved in grass xylan synthesis. Recently, high-density Affymetrix array data for rice has become publicly available, thereby enabling more sensitive co-expression profiling analysis for rice [20].

A number of online tools are available for plant co-expression analysis [21]. Among them, GeneCAT and ATTED-II are databases available for both Arabidopsis and rice co-expression data [22], [23]. ATTED-II currently uses array data from 1388 and 208 GeneChip slides for Arabidopsis and rice, respectively, and genes co-expressed with bait genes are listed according to ‘Mutual Rank’ (MR), which performs significantly better than Pearson's correlation coefficient value [24]. In addition to the co-expression analysis, information regarding subcellular localization can also assist in determining functional associations between proteins [25]. A number of methods have been developed to predict the subcellular location of eukaryotic proteins. These methods can be broadly classified into methods utilizing sorting signals, experimental annotations, and amino acid composition [26]. While these approaches have been used to predict protein localizations in a variety of eukaryotic organelles, they have had limited success when applied to compartments of the endomembrane system [27].

In this study, we performed a comparative analysis of co-expression networks between Arabidopsis and rice, focusing on xylan biosynthesis. From a list comprising 1146 co-expressed genes from Arabidopsis and rice using the ATTED-II database, we identified novel candidates involved in signal transduction, regulation and substrate transport, as well as enzymes directly involved in secondary wall biosynthesis. Furthermore, to predict their subcellular localization, we developed a new algorithm employing a Pfam-based method with experimental data from Arabidopsis. Based on the co-expression analysis and the predictions of subcellular locations, we propose a model of Arabidopsis and rice xylan synthesis and conserved signaling components for secondary cell wall development.

## Results

### Co-expression of three *irx* genes encoding Arabidopsis xylan synthase

**Figure 1:**
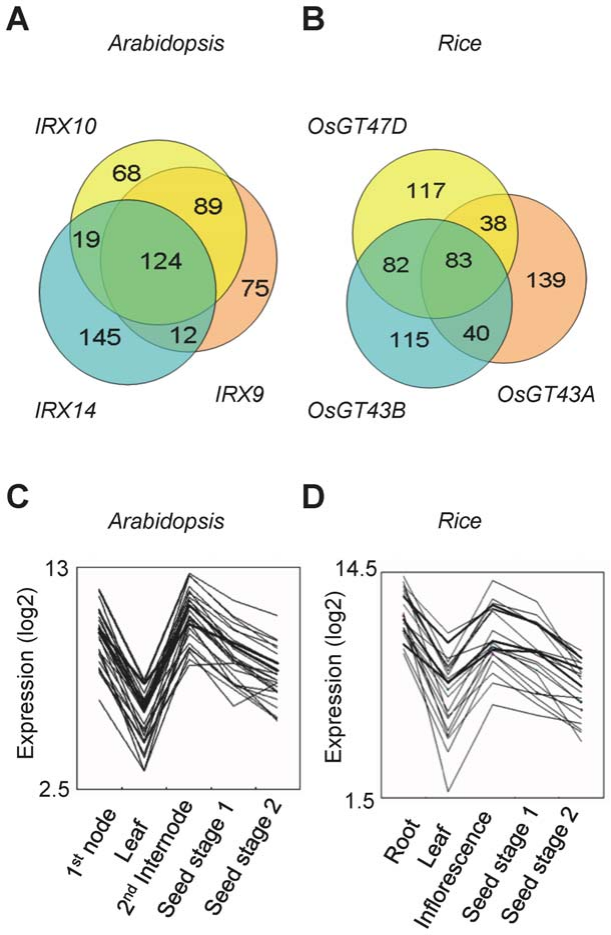
Comparative co-expression analysis between Arabidopsis and rice. (A, B) Venn diagrams of the co-expressed genes with each of the three individual baits from Arabidopsis (A) and rice (B). (C, D) Expression profiles of the 25 genes that most closely match with the baits (thick lines). To confirm co-regulation, the transcriptional expression pattern of the top 25 shared genes of the 3-way intersection from Arabidopsis (A) and rice (B) is plotted. The y-axes show relative gene expression values in base-2 logarithm against the average expression levels of each gene.

In Arabidopsis, genes *IRX9* (At2g37090), *IRX14* (At4g36890), and *IRX10* (At1g27440) are members of the GT43A, GT43B and GT47D subfamilies. The nomenclature used here for the different clades in GT43 and GT47 is according to Arabidopsis [28] and *Brachypodium* [29] studies and differs from that used to designate poplar genes [30]. These *IRX* genes are all expressed in tissues with secondary wall growth and are involved in xylan backbone synthesis [13], [14]. To view the correlation of their expression patterns across many microarray experiments, we performed a scatter plot analysis using CoexViewer based on the 237 data sets related to developmental series in the ATTED-II database (Figure S1) [31]. Scatter plots of pairwise combinations of the three genes showed very similar patterns and strongly correlated expression, whereas the negative control *RALF* gene (At4g15800), which is mainly expressed in tissues with primary wall growth (e.g. rosette leaf), did not show any correlation with *IRX9* expression. To identify other candidate genes likely to be involved in xylan accumulation, we used the CoexSearch tool available at ATTED-II, which uses MR for evaluation of the correlation between two different gene expressions. Table S1 shows the 300 most highly co-expressed Arabidopsis genes obtained for each of the three baits, *IRX9*, *IRX14*, and *IRX10*. Each co-expression list included the three bait genes as strongly co-expressed genes (low MR), and many genes were shared between the three sets of 300 genes, with a total of 124 genes (ca. 23%) shared amongst all three data sets (Table S2; Figure 1A).

The significance of this tight linkage amongst the three genes was further examined by analyzing other members of the Arabidopsis GT43 (*IRX9-L* and *IRX14-L*) and GT47D (*F8H*, *FRA8* and *IRX10-L*) families [32]. The maximum MR range for the 300^th^ gene had weaker values ranging from 400 to 600 (except *FRA8*) when compared to co-expression sets for *IRX9*, *IRX10* and *IRX14* where the maximum MR was less than 400 (Table 1). Surprisingly, Arabidopsis *FRA8*, which appears to be involved in forming the oligosaccharide at the reducing end of xylan, did not tightly co-express with *IRX9, IRX10 or IRX14*. *FRA8* produced a tight network with an MR<192.8 for the 300^th^ most highly co-expressed genes (Table 1), but this network did not overlap considerably with the network defined by xylan backbone synthesis genes *IRX9, IRX10* and *IRX14*. Furthermore, the *FRA8* network did not include the two other known genes implicated in synthesis of the oligosaccharide, i.e. *IRX8* and *PARVUS* [4], [13] while both these genes were co-expressed with *IRX9*/*IRX10*/*IRX14* genes.

**Table 1:** Mutual Rank (MR) of the 300^th^ co-expressed gene to each of the GT43 and GT47D family members.

GT family	AGI (name)	MR of the 300^th^	RAP (Defined name)	MR of the 300^th^
GT47D	**At1g27440 (IRX10)**	**356.9**	**Os01g0926400 (OsGT47D)**	**264.9**
	At2g28110 (FRA8)	192.8	Os01g0926600	264.9
	At5g22940 (F8H)	407.3	Os01g0926700	-
	At5g61840 (IRX10-L)	456.1	Os03g0107900	449.5
			Os02g0520750	-
			Os04g0398600	326.2
			Os10g0180000	226.7
GT43	At1g27600 (IRX9-L)	580.8	Os04g0103100	-
	**At2g37090 (IRX9)**	**295.6**	Os01g0157700	-
	**At4g36890 (IRX14)**	**380.2**	Os01g0675500	244.8
	At5g67230 (IRX14-L)	682.0	Os03g0287800	-
			Os06g0687900	364.7
			**Os04g0650300 (OsGT43A)**	**201.1**
			**Os05g0123100 (OsGT43B)**	**221.3**
			Os05g0559600	400.2
			Os07g0694400	274.4
			Os10g0205300	285.6

The values signify the MR of each of the 300^th^ co-expressed genes with GT43 and GT47D members. Thus, the other 299 co-expressed genes have lower MR values. Low values signify highly correlated expression patterns. The genes shown in bold were used as baits for the final comparative co-expression analysis. The MRs for all genes are listed in Table S1 and Table S3.

### Co-expression analysis of GT43 and GT47D genes in rice

**Figure 2:**
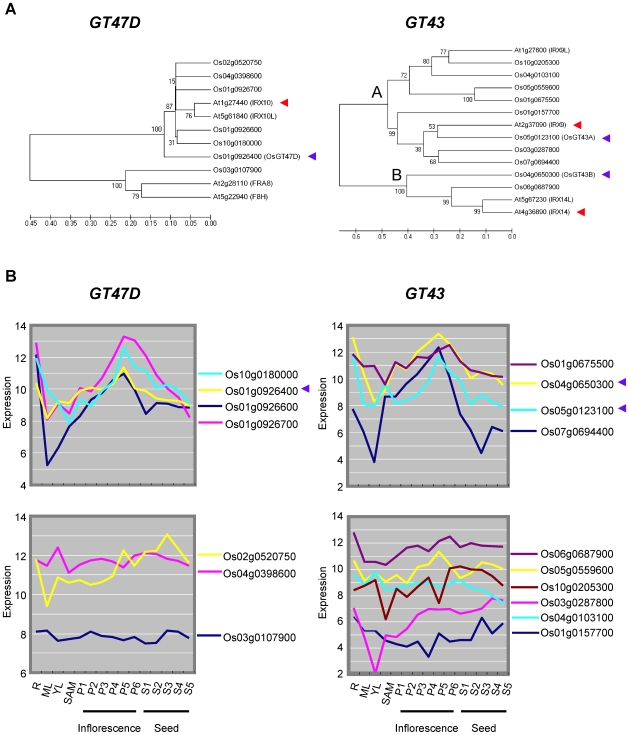
Rice *GT43A, GT43B* and *GT47D* genes. (A) Phylogenic relationship of rice and Arabidopsis genes. Numbers at branches indicate bootstrap values from 500 trials. Phylogenetic tree was built by neighboring-joining method using ClustalW. The *IRX10* and *FRA8* genes in *GT47D*, the *IRX9* and *IRX14* genes in *GT43*, and their rice orthologues are clearly separated into distinct clades. (B) The ‘Mountain type’ expression pattern of the genes showing MR<280 for the 300 most highly co-expressed genes (upper panel). The ‘Flat type’ expression pattern of the genes which showed MR>280 or had no co-expression data available in ATTED-II (lower panel). Os01g0926700 is included in the mountain type because it has a similar profile, although no co-expression data are available for this gene in ATTED-II. The red and blue arrowheads show the genes used as baits for the final comparative co-expression analysis. The y-axes show raw expression values from rice Affymetrix DNA array GSE6893 data [33]. The x-axes show tissue type: R; Root_7d_seedling, ML; Mature_leaf, YL; Young_leaf, P1; Young_inflorescence_P1, P2-P6, Inflorescence stage P2 to P6; S1-S5, Seed stage S1 to S5.

To gain a better understanding of the similarities and difference between xylan gene networks in Arabidopsis and in grasses, we also investigated gene networks in rice. Rice has ten and seven genes belonging to the GT43 and GT47D families, respectively (Table 1). Phylogenic analysis clearly separated the ten GT43 and seven GT47D genes into distinct clades, with six genes in the *IRX10*/*IRX10-L* clade, one gene in the *FRA8/F8H* clade, eight genes in the *IRX9/IRX9-L* clade, and two genes in the *IRX14/IRX14-L* clade (Figure 2A). We examined the expression of the rice GT43 and GT47D genes in different developmental stages using rice Affymetrix DNA array GSE6893 data [33]. Interestingly, the expression patterns could be clearly defined into two distinct groups (Figure 2B). One type of expression profile was strongly dependent on tissue development stage and had high expression levels in tissues associated with secondary wall deposition. The other type of expression profile had relatively constant expression levels. For simplicity, we designate these two patterns ‘mountain type’ and ‘flat type’ expression, based on the appearance in Figure 2B. Most of the genes showing ‘mountain type’ had low range of maximum MR for the 300^th^ gene (Figure 2B, Table 1). The high expression level in tissues with secondary wall formation and the strong co-expression indicate that the ‘mountain type’ genes are the likely homologs implicated in xylan biosynthesis in secondary walls, and hence the functional orthologs of the three Arabidopsis *IRX* genes used for the analysis above.

To select the best rice candidate genes for co-expression analysis we identified the *IRX9*, *IRX10* and *IRX14* homologs that fulfilled the following criteria: 1) ‘mountain-type’ expression profile (Figure 2B), 2) lowest maximum MR in the 300 most highly co-expressed genes and 3) maximum number of shared genes. We selected the *IRX9* ortholog designated ‘*OsGT43A’*, *IRX14* ortholog designated ‘*OsGT43B’*, and *IRX10* ortholog designated ‘*OsGT47D’* and used them as baits for co-expression analysis at ATTED-II (Table 1, Table S3). A large number of shared genes were observed in the pairwise combinations (Figure 1B) with *OsGT47D-OsGT43B* (165 genes), *OsGT47D-OsGT43A* (121 genes), and *OsGT43A-OsGT43B* (123 genes). Combining the three genes, 83 (ca. 14%) of the 300 highest ranked genes were shared (Table S4). These genes included well-known genes such as *BC1* encoding COBL4 and cellulose synthase genes *OsCesA4, OsCesA7* and *OsCesA9*, which are involved in secondary wall synthesis [34], [35]. Figure 1 (C and D) illustrates the transcriptional co-regulation of the top 25 shared genes for both species.

### Development of a Pfam-based predictor for plant endomembrane localization

Knowledge of both co-expression of genes and sub-cellular localization of the corresponding proteins contribute to our understanding of protein function and putative interactions. To date, existing prediction algorithms have been unable to reliably predict localization to the endomembrane system in plants. For other eukaryotes, the pTARGET database employing a genome wide prediction method based on location-specific functional domains currently provides the best prediction for subcellular location in the endomembrane systems [36]. Inspired by this technique we developed a predictor that utilizes Protein functional domain information (Pfam) [37] to predict plant sub-cellular localization. The prediction algorithm required a Pfam training set to establish the baseline distribution of the Pfam domains across multiple subcellular locations. The most comprehensive source of subcellular localization data was obtained from the AmiGO database (http://amigo.geneontology.org). To further improve the robustness of the training set, only 2740 experimentally evidenced (i.e. associated with the ‘IDA’ tag) entries were selected from the 5077 Arabidopsis proteins found in this database. This experimental AmiGO data set was further segmented into groups based upon the subcellular localization associated with the annotation, and Pfam domain information retrieved for each protein. This analysis resulted in data sets that map any given Pfam domain to an experimentally observed subcellular localization. In contrast to pTARGET, the developed prediction algorithm was modified to allow for more than one Pfam domain to contribute to the determination of localization. The pTARGET algorithm bases predictions upon Pfam domains that are uniquely located in specific subcellular compartments. In Arabidopsis, such a method would lead to, at best, 50% of the proteins being correctly localized. By accepting domains that are distributed across different localizations, all loci with Pfam domains can be identified. The trade-off to this method is that the algorithmic detection can become overly broad, and the specificity of the algorithm is lowered. The efficacy of the algorithm is dictated by the size of the training set, and the number of individual Pfam domains that are found in each subcellular compartment (Table S5). Table S6 can be used for the Pfam-based prediction tool. Subcellular localization prediction using Table S6 are performed by finding Pfam domain of a given target protein. For example, a bHLH protein (AT5G48560) has a PF00010 domain and the highest prediction score for PF00010 is 80.9% for nuclear localization. On the other hand, a LRR protein (AT1G67510) contains three Pfam domains, PF000560, PF00069, and PF08263. The highest prediction scores for these Pfam domains are 82.4%, 70.3% and 87.8% for plasma membrane localization, respectively. The final prediction score for this LRR protein for plasma membrane is then calculated as the geometric mean, i.e. the cubic root of (0.824 * 0.703 * 0.878)  = 0.80.

### Characterization of the Pfam-based prediction performance

**Figure 3:**
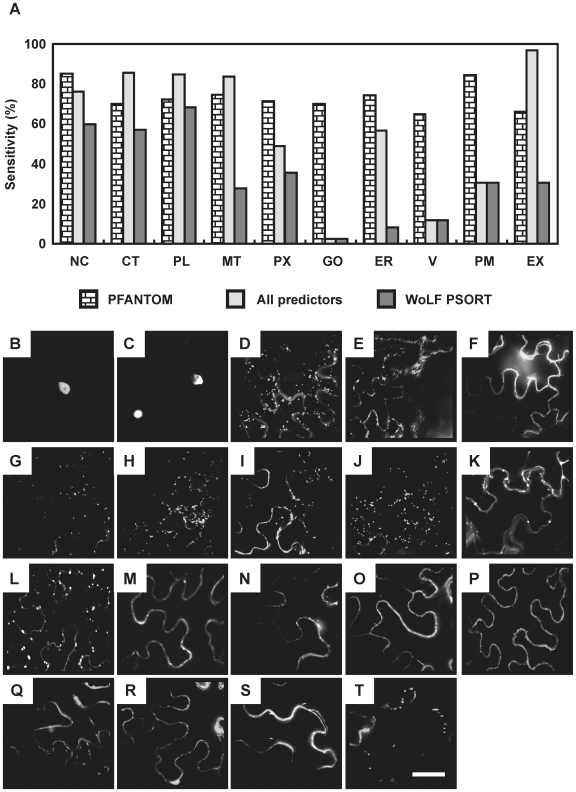
Validation of the PFANTOM method for plant subcellular localizations. (A) Comparison of the prediction performance of Pfam-based prediction (PFANTOM), all predictors by SUBA database, and WoLF PSORT. NC, nucleus; MT, mitochondrion; V, vacuole; PX, peroxisome; ER, endoplasmic reticulum; GO, Golgi apparatus; CT, cytosol; PM, plasma membrane; PL, plastid; EX, extracellular. (B-T) Subcellular localization of transiently expressed YFP-fusion proteins in *N. benthamiana*. (B) bHLH (AT5G48560); (C) SND1 (AT1G32770); (D) Golgi marker (STtmd-GFP); (E) ER marker (GFP-HDEL); (F) Plasma membrane marker (pm-rk); (G) PGSIP1 (AT3G18660); (H) PGSIP3 (AT4G33330); (I) TBL3 (AT5G01360, belonging to DUF231); (J) Unknown protein (AT2G38320, belonging to DUF231); (K) CTL2 (AT3G16920); (L) FLA11 (AT5G03170); (M) FLA12 (AT5G60490); (N) COBL4 (AT5G15630, IRX6); (O) LRR protein (AT1G67510); (P) NCRK (AT2G28250); (Q) RIC2 (AT1G27380); (R) RIC4 (AT5G16490); (S) ROP7 (AT5G45970); (T) ROPGEF4 (AT2G45890). A summary of the localization experiments is shown in Table 2. Scale bar = 20 µm.

Characterization of the Pfam-based predictor was carried out by calculating the sensitivity and specificity of the predictor upon a non-independent set of proteins. Since the algorithm is highly dependent upon the number and uniqueness of Pfam domains to determine localization, the training set was used as a benchmark set to understand how well the algorithm would work in a best case scenario, since all the Pfam domains have already been seen in the training set. Sensitivity across subcellular localizations ranges from 65% (Vacuole) to 85% (Nucleus), while specificity drops down to only 76% (Table S7). To illustrate the threshold-dependency of the algorithm performance, a receiver operating characteristic (ROC) plot was used (Figure S2). Across the different thresholds for including Pfam data in the final calculation, the localization algorithm performs much better than random localization as shown as dotted line on the ROC plots. This algorithm fills a specific need for the predicted localization of endomembrane system proteins. The performance of this algorithm can be readily compared to the best performing predictors as outlined in SUBA, the Arabidopsis subcellular database (Table S7) [38]. SUBA contains pre-calculated localization scores for all Arabidopsis proteins, and the sensitivity and specificity was calculated for the members of the training set for each of the different predictors. SUBA also makes an ‘all predictors’ call using a winner-takes-all localization call to combine the results from multiple individual predictors. A comparison of this integrated ‘all predictors’ call with the Pfam-based predictor indicates that the most significant improvements in sensitivity over current predictors are found for the Golgi, plasma membrane, and vacuole (Figure 3A). In fact only the WoLFPSORT algorithm provides any prediction for these locations, but shows a lower sensitivity rate compared with Pfam-based prediciton. This improvement in localization capability is likely due to the various predictors focusing on properties and datasets not well tailored to the endomembrane system. Based on the improvement in the endomembrane prediction, we named this prediction method PFANTOM (plant protein family information-based predictor for endomembrane) and used Table S6 as the PFANTOM tool.

### Experimental validation of localization predictions

To validate the predictions given by PFANTOM, we selected 16 Arabidopsis proteins predicted by PFANTOM to be located in nucleus, Golgi apparatus, and plasma membrane (Table 2). Thirteen of the Arabidopsis proteins have not previously been examined for their intracellular distribution, whereas the remaining three proteins (ROP7, RIC2, RIC4) have been reported in fluorescent fusion protein experiments to be located in plasma membrane [39], [40] and were included as positive controls. For all 16 proteins the intracellular localization was determined by transiently expressing YFP-fusion proteins in *Nicotiana benthamiana* (Table 2, Figure 3). For comparison we also predicted localization with publically available web-based algorithms (Table 2). As predicted by PFANTOM, two transcription factors, bHLH protein (AT5G48560; Figure 3B) and SND1 (AT1G32770; Figure 3C) showed the YFP signal in nucleus. AT3G18660 (PGSIP1; Figure 3G), AT4G33330 (PGSIP3; Figure 3H), AT5G01360 (DUF231 protein; Figure 3I), and AT2G38320 (DUF231 protein; Figure 3J) showed the YFP signal in small, moving, and oval dots very similar to what was seen with the Golgi marker (ST-tmd-GFP; Figure 3D), and clearly different from the ER marker (GFP-HDEL; Figure 3F), which showed typical network pattern. We selected PGSIP1 and PGSIP3, which were both predicted by PFANTOM to be Golgi localized, although PGSIP1 has been reported to be a chloroplast protein [41] and the Arabidopsis protein does have an N-terminal sequence that appears to fulfill the characteristics of a transit peptide according to the TargetP predictor. Furthermore, we also selected two DUF231 proteins, which were ambiguously predicted to be vacuolar, but which belong to a large family of proteins, several of which are known to play a role in cell wall structure [42]. For the Plasma membrane and/or extracellular localized proteins, YFP fusion proteins of GH19 family and GPI anchored proteins such as AT3G16920 (CTL2; Figure 3K), AT5G03170 (FLA11; Figure 3L), AT5G60490 (FLA12; Figure 3M), AT5G15630 (COBL4, IRX6; Figure 3N), AT1G67510 (LRR protein; Figure 3O), AT2G28250 (NCRK; Figure 3P), AT1G27380 (RIC2; Figure 3Q), AT5G16490 (RIC4; Figure 3R), AT5G45970 (ROP7; Figure 3S) showed the YFP signals as a single layer surrounding the cytoplasm identical to what was observed with a plasma membrane marker (pm-rk; Figure 3E). Interestingly, CTL2 and FLA11 show oval dots in addition to the plasma membrane signal. These dots were larger than for Golgi and we are uncertain what they represent. The YFP signal of AT2G45890 (RopGEF4; Figure 3T) belonging to the GEF family, which is recruited to lipid rafts for small GTPase activation [43], [44] was not uniformly distributed but observed as large dots associated within the plasma membrane, suggesting an interaction with endogenous membrane proteins. The results show that for 11 of the 13 proteins (16 proteins excluding the three positive controls), i.e. all the nuclear and plasma membrane proteins and for the PGSIP proteins, there was agreement between the predicted location by PFANTOM and the observed localization (Table 2).

**Table 2:** Validation of the Pfam prediction of Arabidopsis subcellular localization.

				PFANTOM		Other Prediction Programs				
Experimental Location	Common Name	AGI	Pfam ID	Score (%)	Location	iPSORT	MitoProtII	Predotar	TargetP	WoLF PSORT
**PM**	LRR protein	AT1G67510	PF00560, PF00069, PF08263	80	**PM**			ER	EX	PM
**PM**	NCRK	AT2G28250	PF00069	70	**PM**			ER	EX	unclear
**PM**	ROP7	AT5G45970	PF00071	78	**PM**		MT			CT
**PM**	RIC2	AT1G27380	PF00786	100	**PM**		MT			NC
**PM**	RIC4	AT5G16490	PF00786	100	**PM**		MT	ER	EX	PM
**PM**	ROPGEF4	AT2G45890	PF03759	100	**PM**				PL	NC
**PM**	COBL4 (IRX6)	AT5G15630	PF04833	100	**PM**		MT	ER	EX	V
**PM**	FLA11	AT5G03170	PF02469	82	**PM**		MT	ER	EX	CT
**PM**	FLA12	AT5G60490	PF02469	82	**PM**		MT	ER	EX	PM
**PM**	CTL2	AT3G16920	PF00182	67	**PM**			ER	EX	EX
**NC**	bHLH	AT5G48560	PF00010	81	**NC**					NC
**NC**	SND1	AT1G32770	PF02365	94	**NC**		MT			NC
**GO**	PGSIP1	AT3G18660	PF01501	80	**GO**	CT	MT		CT	unclear
**GO**	PGSIP3	AT4G33330	PF01501	80	**GO**	CT		MT	EX	CT
**GO (PM)**	DUF231 (TBL3)	AT5G01360	PF03005	50/50	**V/PL**				EX	CT
**GO**	DUF231unknown	AT2G38320	PF03005	50/50	**V/PL**		MT	MT	EX	unclear

Sixteen co-expressed genes were selected and intracellular localization was predicted by iPSORT [77], MitoProt II [78], Predotar [79], TargetP.1 [80], and WoLFPSORT [81]. Pfam was the prediction method described in this study, and experimental data are shown in Figure 5. NC, nucleus; MT, mitochondrion; V, vacuole; PX, peroxisome; ER, endoplasmic reticulum; GO, Golgi apparatus; CT, cytosol; PM, plasma membrane; PL, plastid; EX, extracellular.

### Intracellular network for xylan formation in Arabidopsis and rice

**Figure 4:**
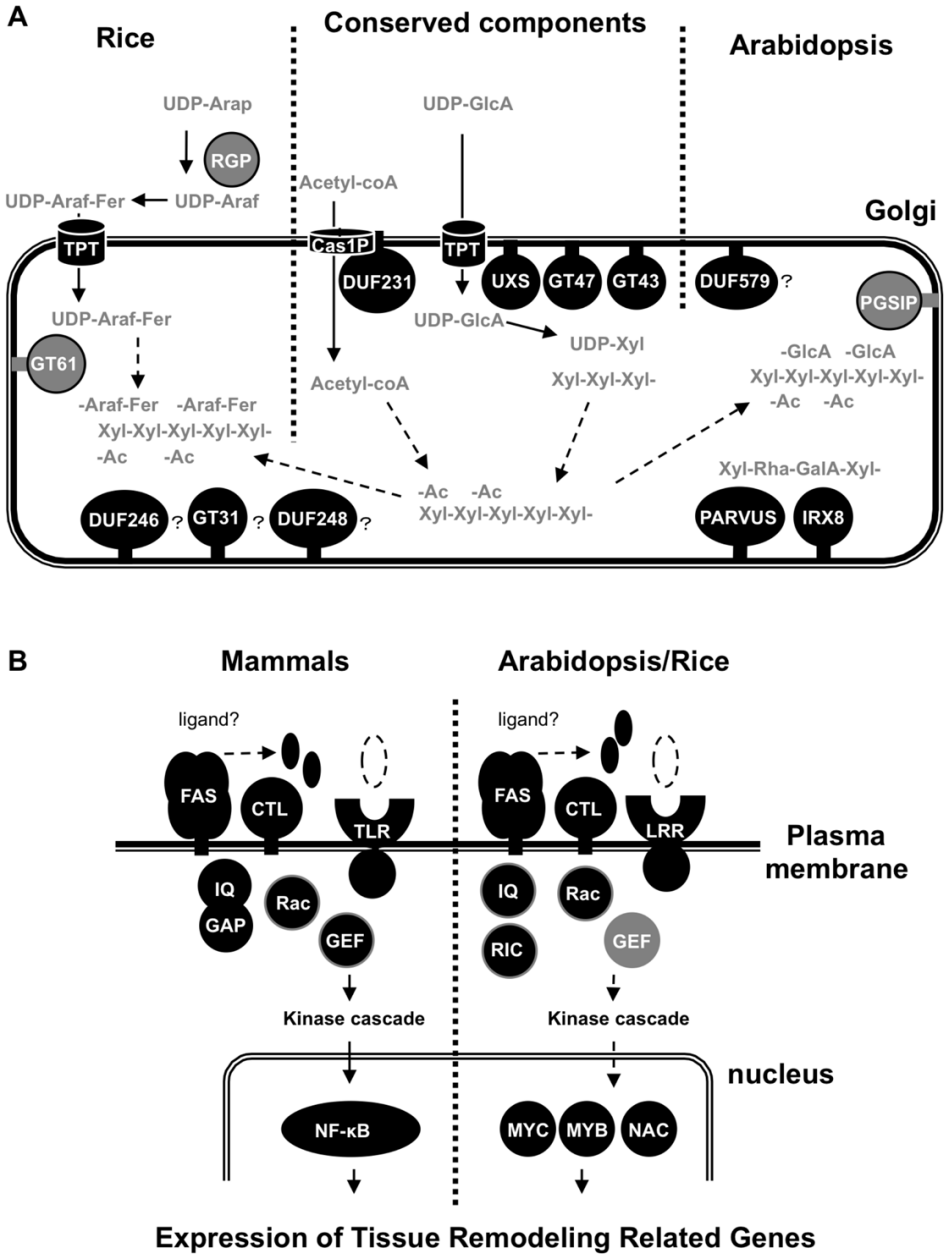
Working model for xylan synthesis and regulatory components for secondary wall development. (A) Xylan synthesis candidates in Golgi apparatus. The components in this model correspond to Table S3. In general only the larger gene family name is shown, except for GT8 and epimerases, which have many members of different molecular function and include PGSIP, IRX8, PARVUS, and UXS. Three gray color components, GT61, RGP and PGSIP proteins, were identified as co-expressed genes unique to Arabidopsis or rice. (B) Signaling and regulatory components on the plasma membrane to nucleus in mammals, Arabidopsis and rice. The components in this model correspond to genes outlined in Table 3. Proteins with a predicted location score more than 0.65 with common functional domain group in mammals and plants were selected as the components on plasma membrane. For the nucleus, proteins with functional domain typically annotated as transcription factor and with a predicted location score greater than 0.8 are shown in this model.

In an attempt to integrate the localization predictions based on Pfam and the co-expression information from Arabidopsis and rice, we examined the Pfam domain information from the intersection sets identified in the previous sections, comprising 124 and 83 genes for Arabidopsis and rice, respectively (Figure S3A, Table S2 and S4). Table S8 outlines the Pfam annotations in the high-ranking co-expression sets showing an average MR of less than 70. Interestingly, many of the Pfam domains were identified in both co-expression sets, suggesting functional components present in both Arabidopsis and rice. Furthermore, several sets of Pfam annotations were unique to either Arabidopsis or rice and likely relate to distinct features of their cell walls. Lastly, to obtain an insight into the putative functional interaction at the subcellular level, the localization of high-ranking co-expressed components in Table S8 were predicted by the PFANTOM (Figure S3B, Table S6). Table 3 outlines the putative intracellular distribution of each Pfam domain from the co-expression set. The majority of proteins from this collection of tightly co-expressed genes could be assigned to three distinct intracellular compartments, the Golgi apparatus (13 Pfam domains), the plasma membrane (21 Pfam domains), and the nucleus (8 Pfam domains). This information assisted modeling of potential interactions in the context of shared subcellular localization. An intracellular working model was constructed outlining common and unique machinery in both Arabidopsis and rice (Figure S3C, Figure 4).

**Table 3:** Analysis of the high-ranking co-expression sets from Arabidopsis and rice using the Pfam-based predictor.

Pfam ID	Predicted	Score (%)	Number of genes		Common protein name	Name used in working model	Related function
			Os	At			
***Golgi apparatus***							
PF03016	GO	100	**4**	**6**	GT47	GT47	Glycosyltransferase
PF03360	GO	100	**4**	**2**	GT43	GT43	Glycosyltransferase
PF01501	GO	80	**3**	**4**	GT8, GAUT/GATL	IRX8; PARVUS	Glycosyltransferase
PF01501	GO	80	**0**	**2**	GT8, PGSIP	PGSIP	Glycosyltransferase
PF01762	GO	67	**3**	**1**	GT31	GT31	Glycosyltransferase
PF04577	no data	0 a)	**3**	**0**	GT61	GT61	Glycosyltransferase
PF03214	GO	100	**2**	**0**	GT75	RGP	UDP-arabinose mutase
PF03141	GO	92	**2**	**1**	Putative methyltransferase	DUF248	methylation
PF04669	no data	0 a)	**1**	**5**	DUF579	DUF579	unknown
PF03138	GO	55	**2**	**2**	DUF246	DUF246	unknown
PF03005	V/PL	50/50 a)	**8**	**5**	DUF231	DUF231	acetylation
PF07779	no data	0 a)	**4**	**1**	O-acetyltransferase-related	Cas1P	acetylation
PF03151	GO	38 a)	**6**	**9**	Triose-phosphate transporter	TPT	NDP-sugar transport
PF01370	GO	13 a)	**4**	**5**	epimerase	UXS	epimerase
***Plasma Membrane (extracellular)***							
PF00071	PM	78	**5**	**7**	ROP, RAB GTPase	Rac	signaling/vesicle
PF00025	PM	57	**2**	**1**	ADP-ribosylation GTPase		unknown/vesicle
PF00612	PM	71	**3**	**7**	IQ protein	IQ	signaling
PF00069	PM	70	**11**	**10**	protein kinase		signaling
PF00560, PF08263, PF00069	PM	80	**3**	**5**	LRR family	LRR	signaling
PF00786	PM	100	**1**	**2**	ROP interactive CRIB	RIC	signaling
PF02469	PM	82	**9**	**4**	Fasciclin-like AGP	FAS	signaling
PF00182	EX	67	**1**	**2**	GH19; chitinase-like	CTL	Glycosyl hydrolase
PF00295	PM	50	**3**	**5**	GH28; Polygalacturonase		Glycosyl hydrolase
PF07983	PM	100	**2**	**3**	GH17; β-(1;3)-glucanase		Glycosyl hydrolase
PF00759	PM	80	**3**	**3**	GH9; cellulase		Glycosyl hydrolase
PF07731, PF00394, PF07732	PM	57	**4**	**6**	putative laccase		lignin formation
PF03552	PM	60	**8**	**3**	cellulose synthase		Glycosyltransferase
PF04833	PM	100	**2**	**1**	COBRA		GPI anchored
PF02298	PM	78	**6**	**4**	plastocyanin-like		GPI anchored
PF00097	PM	51	**12**	**8**	zinc finger		unknown
PF06749	PM	100	**2**	**2**	DUF1218		unknown
PF00190	EX	75	**2**	**3**	Germin-like		unknown
PF07058	PM	67	**0**	**1**	Myosin HC-like		unknown
PF00786, PF00620	PM	71	**0**	**1**	RhoGAP		signaling
PF07320	PM	78	**10**	**0**	Hairpin-induced		unknown
***Nucleus***							
PF00249	NC	96	**6**	**10**	MYB	MYB	Transcription
PF00010	NC	81	**1**	**7**	bHLH (MYC)	MYC	Transcription
PF02365	NC	94	**4**	**9**	NAC	NAC	Transcription
PF00642	NC	83	**2**	**3**	dTIS		unknown
PF04640	no data	0	**1**	**0**	DUF597		unknown
PF00514	NC	54	**3**	**1**	armadillo/beta-catenin repeat		unknown
PF00719	NC	50	**2**	**0**	PRLI-interacting factor		unknown
PF04852	NC	100	**0**	**4**	LSH		unknown
***Other components***							
PF00141	V	38	**11**	**3**	peroxidase		lignin formation
PF00026	EX/PM	33/33	**1**	**1**	aspartyl protease		unknown
PF01419	NC/PL	40/40	**1**	**1**	jacalin lectin		unknown
PF03999	Cytoskeleton	100	**3**	**1**	MAP65-8		unknown
PF04784	PL	67	**1**	**1**	DUF547		unknown
PF00240, PF02179		-	**7**	**1**	Ubiquitin domain		unknown
PF00657	V	64	**2**	**1**	Lipase, GDSL domain		lignin formation
PF06814	V	50	**3**	**1**	Transmembrane receptor		signaling

Groups of protein with the same Pfam domains were found in co-expression dataset. The table shows groups, where at least one gene exhibited an average MR of less than 70. The number of genes corresponding to each Pfam is from the entire co-expression set in Arabidopsis (Table S1) and rice (Table S3). The AGI and RAP codes are listed in Table S8. The predicted location and score are shown according to the Pfam-based predictor. Abbreviations for subcellular compartments are the same as in Table 2.

a)PF01370, PF03005, PF04669, PF03151, PF4577, and PF07779 proteins have been observed in Golgi apparatus (unpublished data) [82]. Based on the references they were categorized into Golgi apparatus, although they showed a low score by PFANTOM.

### PGSIP proteins are putative glucuronyltransferases involved in glucuronoxylan synthesis

**Figure 5:**
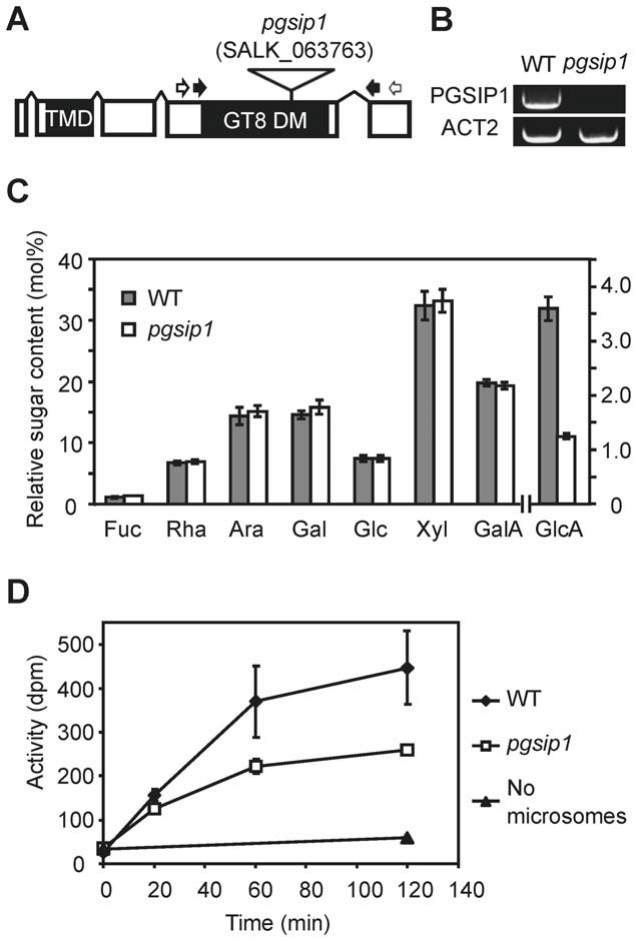
Analysis of an Arabidopsis *pgsip1* mutant. (A) A T-DNA insertion mutant carrying an insertion in the third exon of *PGSIP1* (AT3G18660) was obtained. White arrows indicate primer-annealing sites used for genotyping of the plants, and black arrows indicate primer-annealing sites used for RT-PCR. (B) No functional transcript could be detected by RT-PCR in homozygous individuals. (C) Cell wall material isolated from the first to the third internode of inflorescence stems showed significantly lower contents of GlcA compared to wild type (Col-0) plants (five biological replicates, values are mean ± SD, significantly different at p<10^−6^ (t-test)). (D) Xylan glucuronosyltransferase activity was determined in microsomes prepared from the second internode of inflorescence stems. Data shown are mean ± SD with three biological replicates, each consisting of stem internodes from four plants. The activity is shown in dpm, where the maximal activity determined for Col-0 is about 400 dpm corresponding to 0.15 nmol GlcA incorporated per mg of protein.

The co-expression analysis identified a number of GTs located in the Golgi apparatus (Table 3, Figure 4). Most of these were the *IRX* genes already known to be involved in xylan biosynthesis. The four additional GT groups identified were RGP (GT75) and GT61 in rice, PGSIP1 and PGSIP3 in Arabidopsis, and GT31 proteins in both species but most highly co-expressed in rice. The rice RGPs (UAM1 and UAM3) have been shown to be UDP-arabinose mutases [45]. Since arabinose is abundant in rice xylan but has not been detected in Arabidopsis xylan, the data suggest that GT61 could be xylan arabinosyltransferases, in agreement with earlier speculations [1], [46]. The GT31 proteins do not have an obvious suggested function, but they may be involved in synthesis of arabinogalactan proteins, e.g. the FLA11 and FLA12 proteins that are also seen in the co-expressed data sets. PGSIP1 and PGSIP3 proteins belong to GT8 family which in contrast to GT61 and GT31 contains retaining enzymes [47]. PGSIP proteins are only distantly related to the PARVUS and IRX8 proteins. Therefore, the most obvious function of PGSIP1 and PGSIP3 would be as xylan α-glucuronosyltransferases, given that a major difference between rice and Arabidopsis secondary walls is the 10-fold higher GlcA/Xyl ratio in Arabidopsis (data not shown). To test this hypothesis, we analyzed an Arabidopsis mutant in the *PGSIP1* gene, which is more highly expressed in stems than *PGSIP3*. The *pgsip1* mutant has a T-DNA insertion in the coding region and plants carrying the homozygous insertion were selected by PCR. No functional transcript could be detected in plants homozygous for the insertion (Figure 5). Although no morphological or *irx* phenotype was observed for the *pgsip1* mutant line [11], the monosaccharide composition of cell walls from *pgsip1* stems revealed a highly significant 66% reduction in the content of GlcA compared to the wild type (Figure 5c). None of the other monosaccharides showed a difference. We furthermore tested the xylan GlcA transferase activity in microsomes isolated from stems, using an assay with exogenous xylohexaose as acceptor. The results showed that the GlcA transferase activity in *pgsip1* was only about 50% of the wild type level (Figure 5D). This data strongly supports the hypothesis that PGSIP1 (and likely PGSIP3 as well) is a xylan α-glucuronosyltransferase. Obviously, it will be necessary to substantiate this hypothesis by analysis of an independent allele or complementation of the mutant.

## Discussion

### Two *in silico* approaches; co-expression and localization

To obtain a better insight into the biosynthesis and regulation of xylan across species we extended the strategy of co-expression analysis to both Arabidopsis and rice. Our comparative co-expression analysis used three bait genes in each species and identified both known and novel candidate genes involved in signal transduction, regulation and substrate transport, as well as enzymes directly involved in secondary wall biosynthesis. Previous studies have identified co-expressed genes related to secondary wall formation in Arabidopsis using different transcriptional profiling methods [11], [12], [48], [49]. Persson et al. [12] used regression analysis, Brown et al. [11] analyzed the slope profile using five selected tissue types, Ko et al. [48] identified genes highly expressed in stem, and Mutwil et al. [49] used mutual rank-based correlation matrices (cut-off of 30) for a co-expression network with the secondary wall *CESA* genes. Almost all the components identified in the previous studies are also included in the most highly co-expressed genes in our study (i.e. the 124 Arabidopsis genes in Table S2 include 83% of the genes published in any of the four references. The entire Arabidopsis co-expression list in Table S1 includes 93% of the genes published in any of the four references). Hence, it is clear that it does not make much difference whether the analysis is done with *CESA* genes as in the previous studies or with xylan synthesis *IRX* genes as in our study. Nevertheless, our study led to the identification of many additional genes that were not identified in the previously published studies, including additional kinases, calmodulin binding proteins, MYC and MYB transcription factors, UDP-glucuronic acid decarboxylase (UXS), UDP-glucose 6-dehydrogenase and several glycoside hydrolases (GH) (Table S9). These novel genes were probably identified by this study because our analysis by the MR includes co-expressed genes that show low PCC value [22], [24]. Many of the newly identified genes showed conserved functional domains in both the rice and the Arabidopsis based sets (see below), lending support to the relevance of these genes and our method to identify them. As a further novel strategy in this study, we predicted the subcellular location of the co-expressed proteins. The currently available predictors either could not identify endomembrane proteins or had very low sensitivities for this subcellular location. Consequently we developed a Pfam-based algorithm, PFANTOM that improved sensitivity and specificity (Table S7, Figure S2, Figure 3). However, it should be noted that the ROC plots represent a characterization of a hypothetical best-case scenario based on the distribution of Pfam domains for which experimental data on subcellular location is already available.

Obviously, localization for proteins that do not contain Pfam motifs with localization information cannot be predicted at present. In the lists of co-expressed genes (Table S1 and S3), 85% of Arabidopsis and 84% of rice genes have Pfam information, and 79% of Arabidopsis genes and 76% of rice genes with Pfam domain information can be predicted with PFANTOM. Out of these components, 66% and 75% in Arabidopsis and rice, respectively, are predicted by PFANTOM to be located in the endomembrane compartments. Coverage is still limited for the Golgi apparatus in the Pfam-based predictor, although it exhibits an improved performance over the other available predictors for the Golgi apparatus (Figure 3A). For example, 1) DUF231 proteins, which are localized in Golgi apparatus as shown in Figure 4, were predicted to be localized to the vacuole and the plastid, 2) GT61, DUF579, and Cas1p proteins could not be predicted by the Pfam-based predictor because no published experimental data is available for the associated Pfams (Table 3). More comprehensive studies of the Golgi proteome and/or combining with other prediction algorithm using hydrophobicity [50] would improve the usability of our prediction method. Our new method for the prediction of subcellular locations of plant proteins is robust enough for genome-wide predictions since it does not rely on the presence of signal or target peptides. Therefore, we were able to predict localization for the Arabidopsis and rice co-expression sets to gain further insights into putative functional interactions (Table 3). Interestingly, many of our co-expressed components in secondary wall formation were predicted to be located in the endomembrane system, especially at the plasma membrane and Golgi apparatus, consistent with a role in signal transduction and cell wall formation. The available Golgi proteomic data is quite limited, and this also prevented us from testing the predictor with an independent test set. However, the experimental validation of 11 out of 13 tested proteins was very encouraging. Very recently, a method for subcellular prediction using machine learning and homology has been published and claimed to be efficient at predicting Golgi localization [51]. However, this method cannot predict ER or vacuole, but more importantly, their best classifier could only correctly predict the localization of CTL2, whereas the other 15 proteins we investigated experimentally either had no prediction at all or were incorrectly predicted. We are therefore convinced that our PFANTOM method, in spite of its limitations and simplicity, is better, at least for the analysis of the co-expressed data sets in this study, where proteins located in the endomembrane compartments are highly represented.

### Candidate genes for xylan formation in Golgi apparatus

A working model was developed for proteins identified by co-expression with predicted locations in the endomembrane system (Figure 4A). Genes encoding xylan biosynthesis components were expected to be co-expressed with UDP-GlcA decarboxylase (UXS). Importantly, we identified *AtUXS3* and *AtUXS6* in Arabidopsis and their two orthologs in rice as tightly co-regulated with xylan synthase genes (Table S2, S4, and S9). These proteins had been missed in previously published studies although it is evident that UDP-GlcA decarboxylase must play an important role in secondary wall biosynthesis. Hence, the fact that we find these proteins further supports the relevance of the candidates identified in this study. Further candidates involved in xylan synthesis in the Golgi apparatus, that we identified are nucleotide sugar transporters (TPT) and members of GT8 family (IRX8, PARVUS, PGSIP1, PGSIP3). Although the reducing end structure -Xyl-Xyl-Rha-GalA-Xyl has not been identified in grasses, our rice co-expression profiling identified Os3g0300900 belonging to the GATL clade, the same as PARVUS, and Os3g0211800/Os3g0413400 in the GAUT clade with IRX8. Since IRX8 and PARVUS are apparently involved in forming the reducing-end structure, our findings raise the possibility that rice also has the reducing-end structure. Other unknown proteins specific to plants such as DUF231, DUF246, and DUF579 were also included in co-expressed components in both Arabidopsis and rice. PGSIP1 (At3g18660) and PGSIP3 (At4g33330) and the DUF579 genes (At3g50220, At1g09610, At1g33800, At5g67120, At4g09990) showed stronger co-regulation in Arabidopsis, implying more important roles in Arabidopsis than in rice. PGSIP1 has previously been reported to be located in the plastid and be involved in starch biosynthesis [41]. We have shown here that PGSIP1 and PGSIP3 are clearly located in the Golgi and that PGSIP1 appears to be xylan glucuronosyltransferase (Figure 3G-H, Figure 5). The proteins are unlikely to have a direct function in starch biosynthesis, and in fact we could not observe any difference in starch content in the *pgsip1* mutant by iodine staining of leaves (data not shown). PGSIP1 is a good example how the comparison of wall structure and co-expression patterns between rice and Arabidopsis enabled us to predict a function for PGSIP1, which was in turn experimentally confirmed. Since the genes identified in this study are related to secondary wall formation and not specifically to xylan biosynthesis, we could not a priori assume that e.g. PGSIP1 would have a role in xylan biosynthesis. However, the differences between the two species were most consistent with a role in xylan biosynthesis. We therefore believe that the comparative analysis in the present study is a very powerful tool to form hypotheses that can be tested and yield much more information than analysis of only Arabidopsis as in previous studies.

The localization of the Cas1p-like protein, RWA1 (At5G46340), could not be predicted based on AmiGO data, but the RWA2 protein (At3g06550) has been found in Golgi preparations (H. Parsons and J. Heazlewood, unpublished data). RWA proteins are involved in polysaccharide acetylation (Y. Manabe and H.V. Scheller, unpublished) and have sequence similarity with the C-terminal multimembrane-spanning domain of Cas1p from fungi and animals. Interestingly, recent sequence analysis has shown that the N-terminal domain of Cas1p has similarities with esterases and with DUF231 proteins, while the C-terminal domain has similarity with acetyltransferases [52]. This suggests that DUF231 and RWA proteins in plants exist together in protein complexes, which are likely to catalyze glycan acetylation. We propose that the RWA proteins (4 in Arabidopsis and 3 in rice) are unspecific whereas the DUF231 proteins (47 in Arabidopsis and 59 in rice) confer the specificity for particular polysaccharides. The presence of RWA1 and DUF231 proteins in the co-expressed sets may suggest their involvement in xylan acetylation, but they may have other roles in secondary walls as suggested by analysis of DUF231 mutants that are deficient in cellulose [42].

The co-expressed genes also include GT31 proteins (Table 3, Figure 4), which may be involved in arabinogalactan biosynthesis. Interestingly, many of the rice genes in the families of galactosyltransferase (GT31), putative methyltransferase (DUF248) and Golgi unknown protein (DUF246) were identified as components showing strong co-regulation, compared with Arabidopsis, suggesting a more important role in rice cell walls and potentially related to the structural differences between rice and Arabidopsis walls. Furthermore, rice co-expression profiling showed that 18 genes comprising 5 Pfams are specific to rice, with no orthologs in the Arabidopsis co-regulation network. They include RGPs (UAM1 and UAM3), which have UDP-arabinose mutase activity [45], and several GT61 proteins. The GT61 proteins are good candidates for xylan arabinosyltransferases [1], [46]. Mitchell et al. [19] identified members of the BAHD acyltransferases as candidate feruloyl transferases, and recent work supports their involvement in rice xylan feruloylation [53]. The BAHD proteins are cytoplasmic and this is consistent with a role in feruloylation of a cytoplasmic intermediate and not a direct feruloylation of xylan [1], [54]. We do not find BAHD family genes co-expressed with all three baits in rice, but Os06g0595800 and Os02g0483500 are BAHD family genes that are co-expressed with two of the baits, *OsGT43B* and *OsGT47D* (Table S3). Hence, these two BAHD members are good candidates for feruloyltransferases involved in xylan biosynthesis.

### Highlights on signaling and regulatory components

The co-expression analysis revealed a large number of highly co-expressed plasma membrane associated proteins and transcription factors (Table 3). In general, orthologous or very similar proteins were found in both the rice and Arabidopsis co-expressed gene sets. Many of the identified proteins belong to protein families well known to participate in signal transduction. Notably, co-expression of entire gene sets related to GTPase signal cascade were conserved in both Arabidopsis and rice. GTPase signal cascade such as LRR receptor kinase, Rop/Rac GTPase, RICs, RopGEFs, and IQ domain protein resemble components known from other signal transduction pathways in mammals where such pathways are understood in more detail than in plants. As an example we have shown components of the mammalian TLR2 signaling cascade in Figure 4B. TLR2 contains an extracellular LRR domain that is critical for transmitting the peptideglycan, lipopeptide, and chitin signal across the cell membrane to initiate innate immunity response against pathogens [55], [56]. An adaptor molecule, MyD 88, associated with the toll/interleukin-1 receptor (TIR) intracellular domain of TLR2, recruits PI3K kinase, which is also regulated by Rho/Rac GTPase via RhoGEF protein [39], [55], [57]. The TLR complex, consisting of MyD88, PI3K kinase, and Rho/Rac GTPase activates a MAP kinase cascade, which leads to the activation of transcription factors including NF-κB [55]. Recent studies show that TLR2 and another receptor, TLR4, could also percept endogenous ligands and lead to not only immune response but also tissue remodeling especially for neurogenesis [58]. Similar to TLRs, the extracellular LRR domain of plant LRR kinase may recognize small molecules such as peptides and saccharides, while the intracellular kinase domain of the LRR protein transduces the signal to kinase cascades when activated by Rop/Rac GTPase proteins. The Rop/Rac signaling activity is regulated by RICs and RopGEFs [59], [60]. AtRAC2/ROP7 is specifically expressed during late stages of xylem differentiation in Arabidopsis [39]. This signaling by AtRAC2/ROP7 might be mediated by co-expressed IQ domain proteins in our list based on the report that human IQGAP protein interacts with Rho/Rac GTPase [61]. This result suggests that they are key regulatory pathways during secondary wall development and can be crucial for the signaling perception.

An important question concerns the actual signals that trigger the pathway. Strongly co-expressed components such as fasciclin-like arabinogalactan proteins (FLA11, FLA12) and chitinase-like protein (CTL2) were located to plasma membrane (Table 2). In support of a role of these proteins in secondary wall development, high expression levels of *CTL* and *FLA* genes were also found in development of poplar tension wood and cotton fiber [62], [63], [64], [65]. Two similar protein families, namely the fasciclin domain containing protein TGFBI (βig-H3), and chitinase-like proteins, CHI3L1 and CHI3L2, are present in mammals, and recent Massively Parallel Signature Sequencing (MPSS) analysis show similar expression pattern in brain cancer cells [66]. Another mammalian fasciclin domain containing protein, Stabilin-1, is receptor protein and has been reported to interact with chitinase-like protein SI-CLP [67]. Both SI-CLP and the plant chitinase-like proteins including CTL1 (At3g16920) and CTL2 (At1g05850) lack a chitin biding domain and catalytic residues involved in chitin hydrolysis and appear to have no chitinase activities [30], [67], [68]. Based on these conserved characteristics of the chitinase-like proteins, i.e. lacking chitin-binding domain and chitinase activity, and transcriptional co-regulation with fasciclin domain proteins, chitinase-like proteins could bind with fasciclin domain proteins in plants as well as mammals and might lead to ligand-receptor signaling for the GTPase cascade [67] (Figure 5b). As the final components in the signal transduction pathways, we find several transcription factors such as MYB, MYC, and NAC, which may be activated by the kinase cascades and/or the calcium signaling and turn on downstream target genes, e.g. genes involved in cytoskeleton organization and encoding cellulose, lignin and xylan biosynthetic enzymes [69], [70], [71]. The simple model outlined in Figure 4B will clearly need modification as additional components in the signal transduction pathways are identified and as the hypothesized interactions are experimentally tested. Nevertheless, such models are useful frameworks for developing hypotheses that can be tested.

### Conclusion

By the combined *in silico* approaches of expression profiling and localization prediction, we identified putative components of the intracellular network related to xylan synthesis and secondary wall development and proposed models for their function and interactions. Many of the components are identified in both Arabidopsis and rice, giving confidence that they have important roles in the functional network. The analysis enabled us to hypothesize a function of PGSIP proteins as xylan glucuronosyltransferases that was subsequently experimentally verified. To obtain direct evidence of the role of the other candidate genes in secondary wall formation, future work will involve confirmation of protein-protein interactions, and determination of enzymatic activity of the biosynthetic enzymes.

## Methods

### Co-expression analysis and assessment of the gene function

Co-expression information was obtained from the ATTED-II database (http://atted.jp). Source of GeneChip data in ATTED-II version 5.5 are the 1388 array slides from the 58 experiments on each developing stage, biotic and abiotic treatment. Scatter plots of co-expression of two genes were made with CoexViewer available at the ATTED-II database [31]. ATTED-II provides the top 300 genes co-expressed with bait genes in both Arabidopsis and rice. We used three bait genes for each species, obtained the MR for each gene, and calculated the average MR as the geometric mean of the three individual MR. Transcript level information during developmental stage in Arabidopsis and rice were obtained from Arabidopsis Affymetrix DNA array data available from AtGeneExpress at TAIR (http://www.arabidopsis.org) and rice Affymetrix DNA array data GSE6893 available from Rice array database (http://www.ricearray.org) [20]. The Pfam database (ver. 24.0) has a collection of 7677 unique protein functional domains based on Hidden Markov Models (http://pfam.wustl.edu) [37]. Pfam domain information of whole genome in Arabidopsis was downloaded from TAIR and for rice from the rice genome annotation project (http://rice.plantbiology.msu.edu)[72].

### Pfam domain profiling of Arabidopsis proteins

To develop the Pfam domain-based algorithm in plants, we downloaded Arabidopsis gene product information from the AmiGO database (http://amigo.geneontology.org) for the following localization terms; GO:0005634 (3012 proteins; nucleus), GO:0005739 (1310 proteins; mitochondrion), GO:0005773 (621 proteins; vacuole), GO:0005777 (201 proteins; peroxisome), GO:0005783 (407 proteins; endoplasmic reticulum), GO:0005794 (238 proteins; Golgi apparatus), GO:0005829 (669 proteins; cytosol), GO:0005886 (2236 proteins; plasma membrane), GO:0009504 (18 proteins; cell plate), GO:0009536 (3724 proteins; plastid), and GO:0048046 (333 proteins; extracellular). To remove uncertain localization annotations such as ‘by similarity’ or ‘probable’, we restricted the gene products to 4422 Arabidopsis genes with the IDA evidence code, which indicates that the annotation is derived from experimental data. Since a protein can have multiple Pfam domains, this set of 4422 genes encoded a total of 6141 Pfam domain annotations consisting of 1781 different Pfam domains (Table S5). In order to make predictions about the location of un-localized proteins, a reference data set was established from the AmiGO-derived data, which captured the distribution of Pfam annotations across the different localization GO terms. Since it is possible in the training set for a single Pfam to be annotated to more than one subcellular localization, we define a localization ratio for a single Pfam as the percentage of time the Pfam annotation is seen in a given localization. For a new protein, a score (valued from 0–100%) for a single localization can be obtained by calculating the geometric mean of the localization ratio for each of the Pfam domains that it is annotated with. By calculating this score for all localizations, and selecting the localization with the highest score, it is possible to suggest the localization for a protein.

### Cloning and transient expression of proteins

All clones used in this study were constructed using Gateway™ technology (Invitrogen). The Entry clones were obtained via BP-reaction in pDONR-Zeo (for Golgi proteins) or through TOPO-reaction using the pENTR/D-TOPO vector (for plasma membrane and nuclear proteins). The genes were cloned using cDNA from Arabidopsis stem as template. The reverse primers contained no stop codon to enable C-terminal fusions. Sequences of forward and reverse primers can be sent on request. All Entry clones were verified by restriction analysis and sequencing.

The binary vectors for expression of the N-terminal YFP fusion proteins under the control of 35S promoter were constructed via LR-reaction using the corresponding Entry clones. The full-length genes were cloned into the destination vectors pEarleyGate 101 [73]. Marker proteins for ER (GFP-HDEL), Golgi (STtmd-GFP), and plasma membrane (pm-rk) have been described previously [74], [75]. The gene encoding p19 protein from tomato bushy stunt virus was used to suppress gene silencing. All vectors were used to transform *Agrobacterium tumefaciens* strain C58-1 pGV3850. Prior to leaf infiltration the bacteria were resuspended in AS-medium (10 mM MgCl_2_, 150 µM acetosyringone, 10 mM MES pH 5.7) to OD_600_ 0.5. *Agrobacterium* strains containing the YFP constructs and the p19 silencing plasmid were mixed 1∶1 and co-infiltrated into leaves of 3–4 week old *N. benthamiana* plants. Abaxial epidermis of infiltrated leaves was assayed for fluorescence by confocal laser-scanning microscopy 2–3 d post infiltration.

### Confocal Microscopy

A Leica confocal microscope (Leica Microsystems) was used for confocal laser-scanning microscopy. All images were obtained with 63× magnification and a glycerol-immersion objective. GFP and YFP channels were acquired by simultaneous scanning using 488-nm laser lines for excitation; signals were detected between 500 and 530 nm. Images were processed using the Leica Confocal Software (Leica Microsystems) and Adobe Photoshop 7.0.

### Mutant analysis and glycosyltransferase assay

The T-DNA insertion mutant in the *PGSIP1* gene (SALK_063763) was obtained from the Arabidopsis Biologial Resource Center, Ohio. Plants were grown under short day conditions (8 h photoperiod) in growth chambers for 6 weeks before they were transferred to a growth room with a long-day regime (16 h photoperiod). After 14 days growth under long-day conditions the first to third internodes were harvested and cell walls prepared, hydrolyzed with TFA and subsequently analyzed by HPAEC for monosaccharide composition as previously described [76].

Xylan glucuronosyltransferase activity in microsomes prepared from stems was determined essentially as described [10], using 3.7 µM UDP-^14^C-d-GlcA (740 Bq per reaction, MP Biomedicals, Solon, Ohio), 50 µM unlabeled UDP-d-GlcA, and 6 µg xylohexaose (Megazyme, Bray, Ireland) as acceptor in a 30 µl reaction volume. Products were separated by paper chromatography and analyzed by liquid scintillation counting according to Lee et al. [10].

For RT-PCR, total RNA was isolated from frozen stem tissue using the Plant RNeasy Mini kit (Qiagen) according to the manufacturer's instructions. First-strand DNA synthesis was performed with oligo(dT) anchor primer and Superscript III reverse transcriptase (Invitrogen) according to the manufacturer's instructions. Two µL were used as template for PCR using the primers 5′-GTTTACGTCTGCGGTGCAAT-3′ and 5′-AATTATTGCGTCACAAGTTATGG-3′ to amplify PGSIP1 cDNA and 5′-CTCAAAGACCAGCTCTTCCATC-3′ and 5′-GCCTTTGATCTTGAGAGCTTAG–3′ to amplify ACT2 cDNA. The PCR program consisted of 2 min at 95°C, followed by 30 cycles of 20 s at 95°C, 30 s at 49°C, and 1 min 15 s at 72°C, with a final extension step of 10 min at 72°C. PCR products were visualized on 0.8% agarose gels.

## Supporting Information

Figure S1 **Expression correlation among the three IRX genes.** (A–D) Scatter plot analysis between *IRX9* and *IRX10* (A), *IRX9* and *IRX14* (B) *IRX10* and *IRX14* (C), *IRX9* and a non‐co‐expressed gene (At4g15800) as negative control (D).
(TIF)

Figure S2 **Comparison of the prediction performance of different subcellular locations using ROC plots.** Dotted line shows a random assignment.
(TIF)

Figure S3 **Flowchart of data processing and analysis in this study.** (A) Co-expressed gene information from Arabidopsis and rice to the Pfam functional domain information. (B) Arabidopsis gene product information with subcellular localization data to the Pfam functional domain information. (C) Integration of the conserved functions across the species from (A) and subcellular localization information form (B) with the Pfam domains. The integrated information leads to the intracellular working model across the species. The working model was validated by the fluorescence protein experiments, knock-out mutant analysis, and/or enzyme assay.
(TIF)

Table S1 **300 co-expressed Arabidopsis genes listed by three baits, *IRX9* (At2g37090), *IRX10* (At1g27440) and *IRX14* (At4g36890).**
(XLS)

Table S2 **The 124 shared Arabidopsis genes.**
(XLS)

Table S3 **300 co-expressed rice genes listed by three baits, *OsGT43A* (Os05g0123100), *OsGT43B* (Os04g0650300) and *OsGT47D* (Os01g0926400).**
(XLS)

Table S4 **The 83 shared rice genes.**
(XLS)

Table S5 **Distribution of Pfam domains across sub-cellular localizations.**
(XLS)

Table S6 **Localization scores used for PFANTOM tool.**
(XLS)

Table S7 **The specificities and sensitivities of each predictor in ten subcellular compartments.**
(XLS)

Table S8 **The Pfam information of the high-ranking co-expression sets.**
(XLS)

Table S9 **Comparison of the co-expressed genes presented in this study and previously published studies.**
(XLS)
