# Supplementary figures and images for: An Integrative Approach to the Identification of Arabidopsis and Rice Genes Involved in Xylan and Secondary Wall Development
Source: PLoS One. 2010 Nov 23;5(11):e15481. doi: 10.1371/journal.pone.0015481 (PMC2990762; doi:10.1371/journal.pone.0015481)

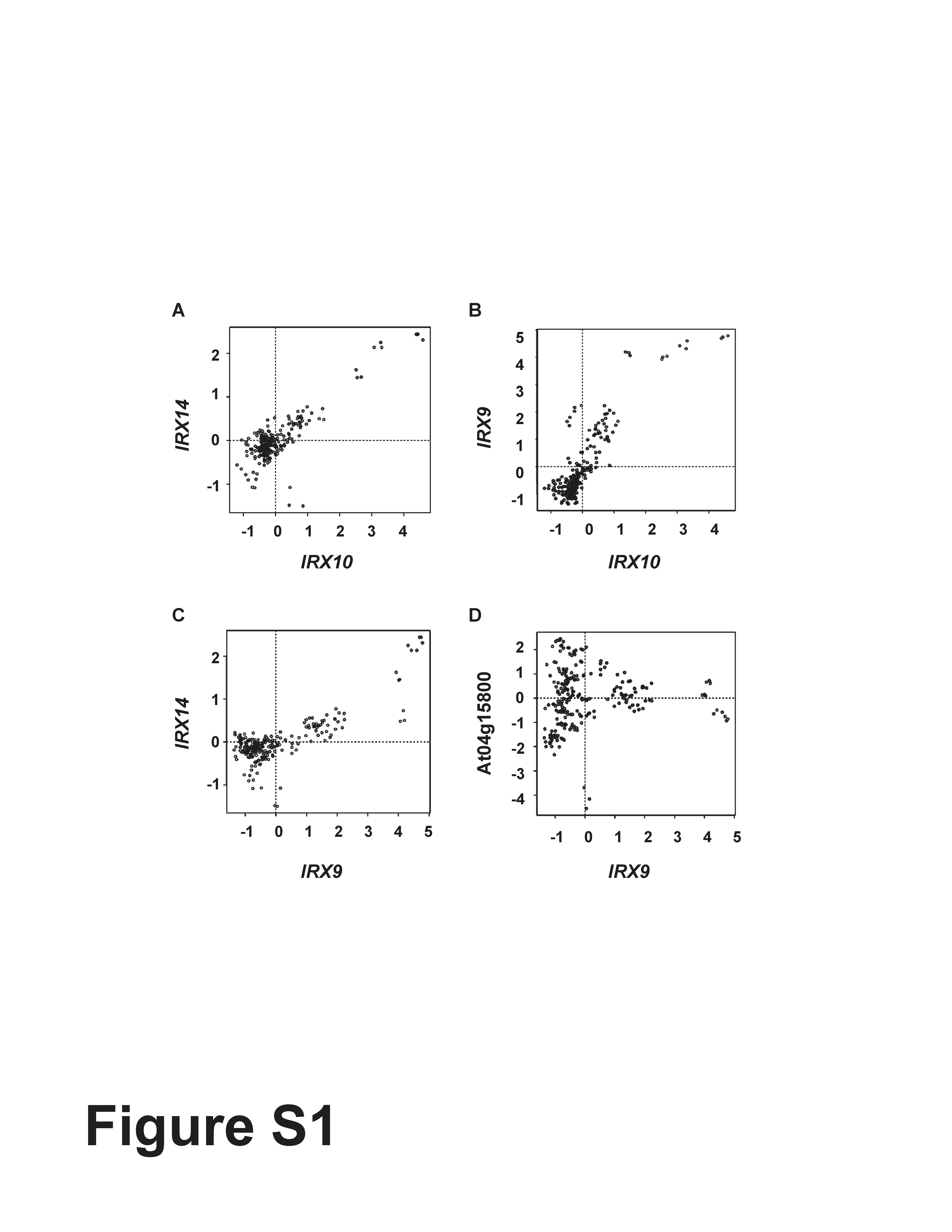

Supplement: Figure S1 — Expression correlation among the three IRX genes. (A–D) Scatter plot analysis between IRX9 and IRX10 (A), IRX9 and IRX14 (B) IRX10 and IRX14 (C), IRX9 and a non‐co‐expressed gene (At4g15800) as negative control (D). (TIF) [file pone.0015481.s001.tif]

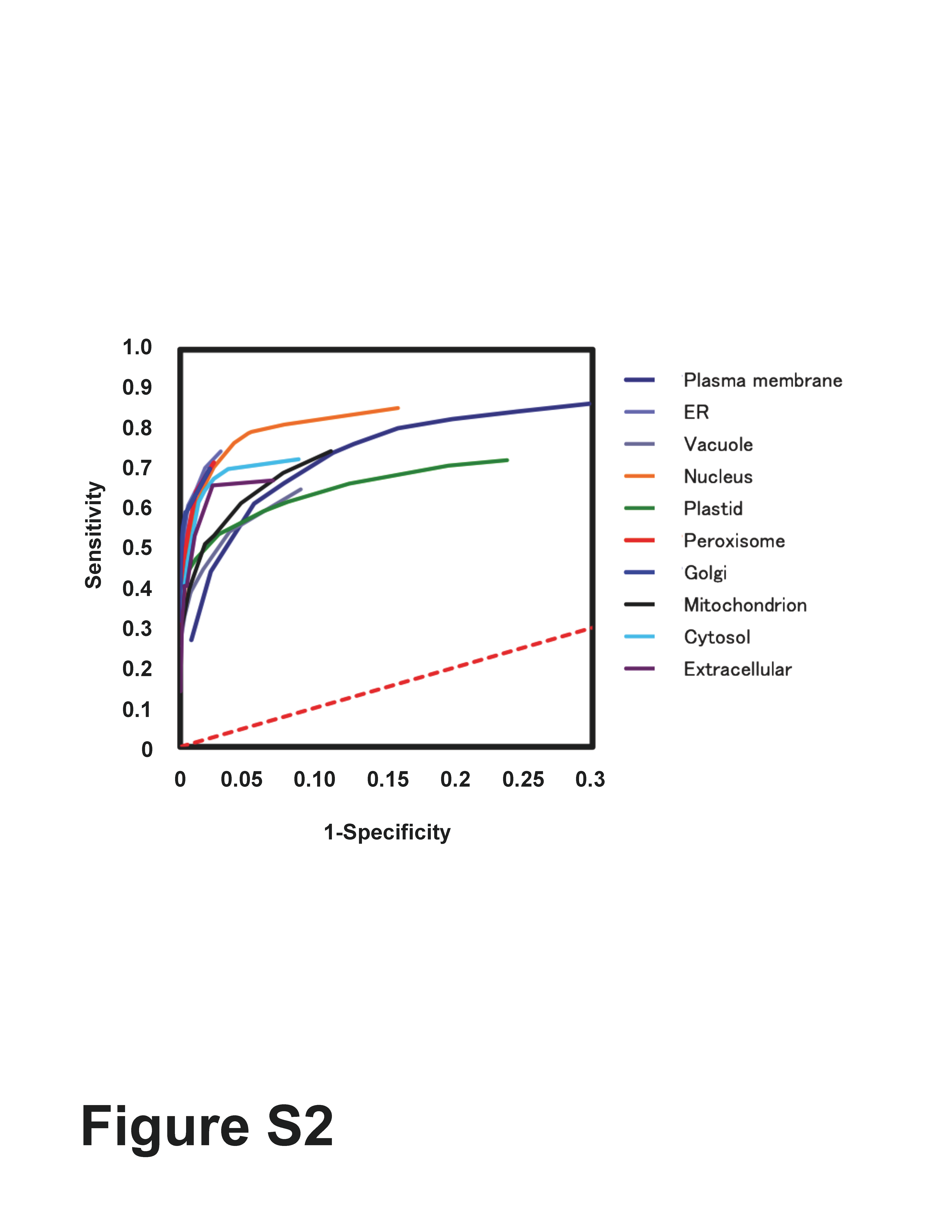

Supplement: Figure S2 — Comparison of the prediction performance of different subcellular locations using ROC plots. Dotted line shows a random assignment. (TIF) [file pone.0015481.s002.tif]

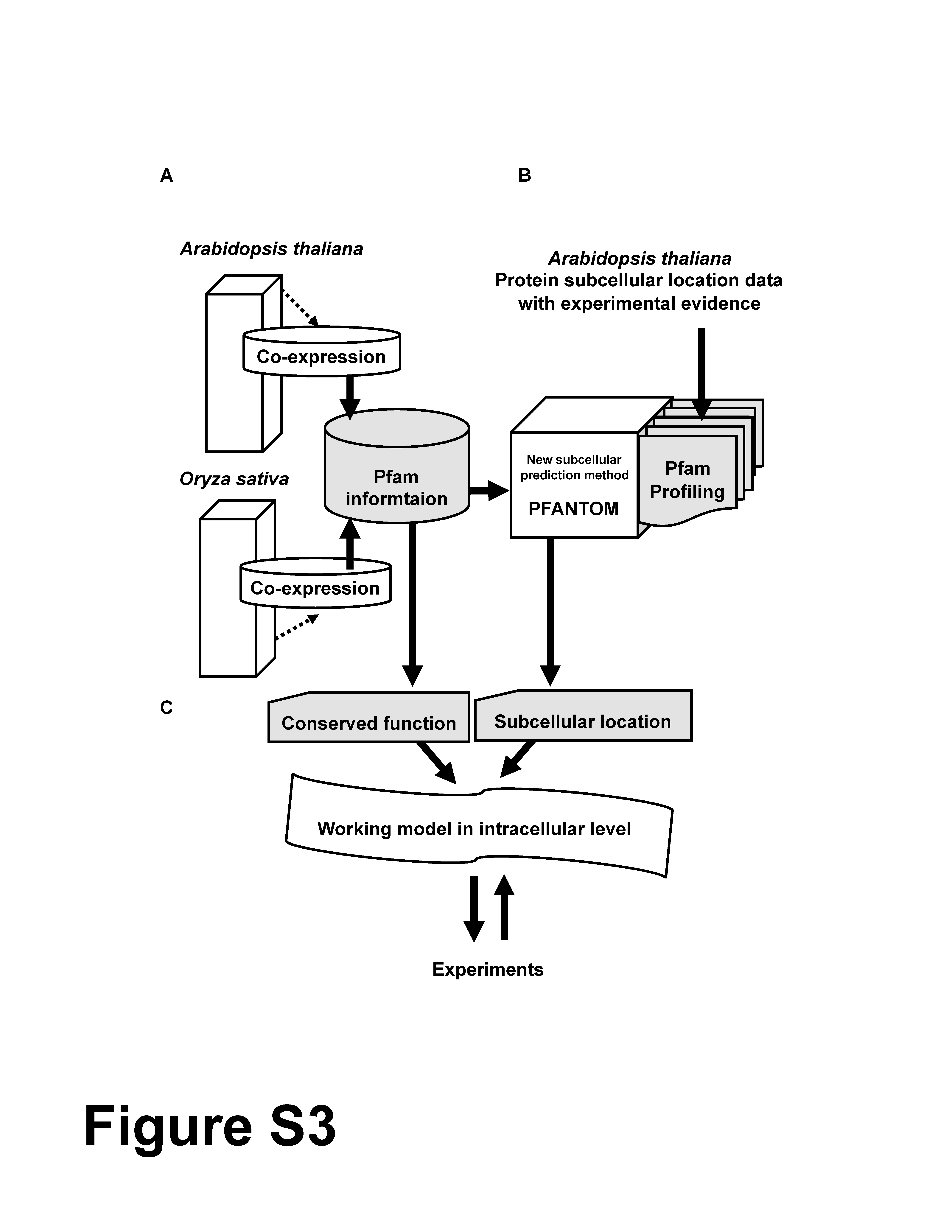

Supplement: Figure S3 — Flowchart of data processing and analysis in this study. (A) Co-expressed gene information from Arabidopsis and rice to the Pfam functional domain information. (B) Arabidopsis gene product information with subcellular localization data to the Pfam functional domain information. (C) Integration of the conserved functions across the species from (A) and subcellular localization information form (B) with the Pfam domains. The integrated information leads to the intracellular working model across the species. The working model was validated by the fluorescence protein experiments, knock-out mutant analysis, and/or enzyme assay. (TIF) [file pone.0015481.s003.tif]
